# Estrogen receptor beta reduces colon cancer metastasis through a novel miR-205 - PROX1 mechanism

**DOI:** 10.18632/oncotarget.9895

**Published:** 2016-06-07

**Authors:** Trang Nguyen-Vu, Jun Wang, Fahmi Mesmar, Srijita Mukhopadhyay, Ashish Saxena, Catherine W. McCollum, Jan-Åke Gustafsson, Maria Bondesson, Cecilia Williams

**Affiliations:** ^1^ Center for Nuclear Receptors and Cell Signaling, Department of Biology and Biochemistry, University of Houston, Houston, TX, USA; ^2^ Department of Biosciences and Nutrition, Karolinska Institutet, Huddinge, Sweden; ^3^ Department of Pharmacological and Pharmaceutical Sciences, University of Houston, Houston, TX, USA; ^4^ Science for Life Laboratory, School of Biotechnology, KTH The Royal Institute of Technology, Solna, Sweden

**Keywords:** colorectal cancer, PROX1, estrogen receptor, microRNA, metastasis

## Abstract

Colon cancer is a common cause of cancer death in the Western world. Accumulating evidence supports a protective role of estrogen via estrogen receptor beta (ERβ) but the mechanism of action is not known. Here, we elucidate a molecular mechanism whereby ERβ represses the oncogenic prospero homebox 1 (PROX1) through the upregulation of miR-205. We show that PROX1 is a potential target of miR-205 and that in clinical specimens from The Cancer Genome Atlas data, ERβ and miR-205 are decreased in colorectal cancer tissue compared to non-tumorous colon, while PROX1 levels are increased. Through mechanistic studies in multiple colorectal cancer cell lines, we show that ERβ upregulates miR-205, and that miR-205 targets and represses PROX1 through direct interaction with its 3′UTR. Through the generation of intestine-specific ERβ knockout mice, we establish that this pathway is correspondingly regulated in normal intestinal epithelial cells *in vivo*. Functionally, we demonstrate that miR-205 decreases cell proliferation and decreases migratory and invasive potential of colon cancer cells, leading to a reduction of micrometastasis *in vivo*. In conclusion, ERβ in both normal and cancerous colon epithelial cells upregulates miRNA-205, which subsequently reduces PROX1 through direct interaction with its 3′UTR. This results in reduced proliferative and metastatic potential of the cells. Our study proposes a novel pathway that may be exploited using ERβ-selective agonists and/or miR-205-replacement therapy in order to improve preventive and therapeutic approaches against colon cancer.

## INTRODUCTION

Colorectal cancer remains the third leading cause of cancer death in the United States claiming approximately 50 000 lives yearly [1]. Because of its slow development and our capacity for early detection using endoscopy, there should be excellent prospect for preventive therapies. However, current preventive approaches of surgical removal of polyps and long-term aspirin treatment are not sufficient, and a truly preventive or targeted therapy remains to be developed. Epidemiological studies indicate a role for estrogen in protecting against colorectal cancer [2–5]. Patients with inflammatory bowel syndrome are at high risk of developing colorectal cancer, and within this group men are 60% more likely to develop colorectal disease than women [6]. Experimentally, 17β-estradiol (E2) has been demonstrated to reduce the formation of preneoplastic lesions in mice [7]. The effect of estrogen is mediated by estrogen receptors (ERs): ERα (ESR1) and ERβ (ESR2). In the normal colon, ERβ is the predominate ER [8, 9]. Its expression decreases when colon cancer progresses, and this correlates with more advanced Dukes' staging [9–12]. A lack of ERβ in the tumor is independently associated with poor survival in patients [13, 14], and a polymorphism in dinucleotide (CA) repeats of the ERβ gene has been associated with increased colorectal cancer risk in women [15]. ERβ has further been demonstrated to have a protective role against colorectal cancer in animal models [16–20]. While both ERs are activated by estrogen, their ligand-binding domains differ slightly and selective ER modulators (SERMs) has been designed to preferentially activate either ER. There is thus a potential for ERβ-selective agonists as a chemopreventive approach against colon cancer development. However, the underlying molecular mechanism for this protective effect and the function of ERβ in the colon is not understood.

We have previously found that the level of the microRNA (miRNA) miR-205, is increased upon expression of ERβ in SW480 cells [21]. The function of miR-205 is relatively unknown in colon cancer, but low levels correlate with increased invasion into lymphatic vessels [22]. miRNAs acts by regulating protein levels through complementary binding between the miRNA 5′-sequence and the 3′-untranslated region (3′UTR) of target mRNAs. We have characterized the impact ERβ has on the transcriptome of colon cancer cells [23], and in particular, we noted a downregulation of prospero homebox 1 (PROX1) in the cancer cell line SW480 [23]. PROX1 is known to be upregulated in colon adenomas and is associated with a poor grade of tumor differentiation and with worse outcome, especially in women [24]. It is associated with the transition from benign adenoma to carcinoma *in vivo,* and its silencing reduces size and incidence of human colorectal tumor xenografts [25]. PROX1 also promotes epithelial-to-mesenchymal transition (EMT) in colon cancer cells [26]. In this study, we describe a putative miR-205 binding site in the 3′UTR of *PROX1,* and we propose that this is a key mechanism behind the estrogen-mediated colorectal cancer-protective effect. We test whether ERβ silences PROX1 expression through the upregulation of miR-205 and explore the functional effects of this regulation.

## RESULTS

### Loss of ERβ is accompanied by loss of miR-205 and increased PROX1 levels in primary colorectal cancer specimens

**Figure 1:**
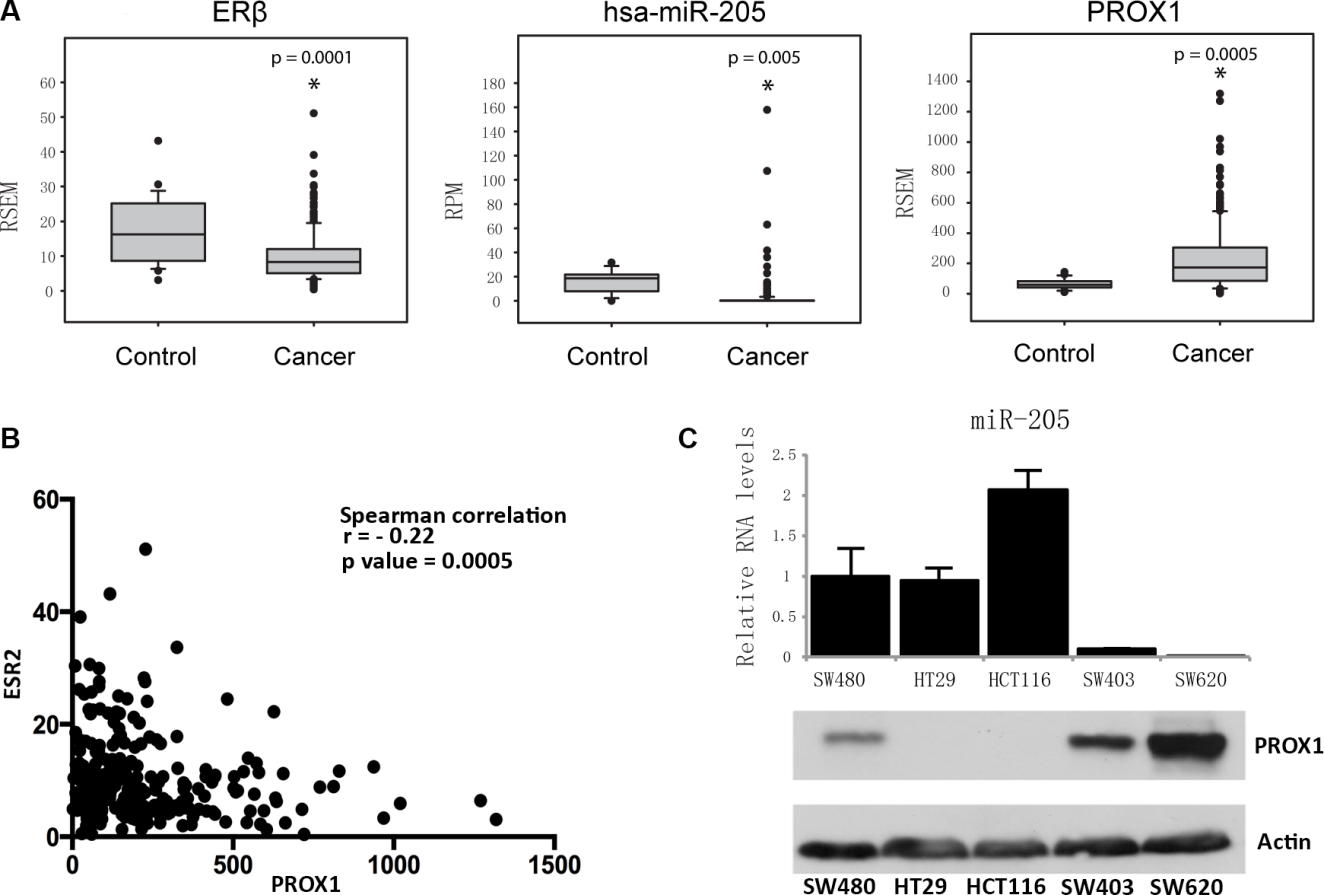
Expression of ERβ, miR-205, and PROX1 in human colon tissues and cells (**A**) In primary colorectal tumor samples ERβ mRNA levels are decreased compared to non-tumorous tissue. This is accompanied by decreased miR-205 and increased *PROX1*. Data were collected from The Cancer Genome Atlas (TCGA), and illustrated using box plots. RSEM was used for transcript quantification of mRNAs. RPM indicates reads per million, for miRNAs. (*P* < 0.05, Student's *t*-test) (**B**) Spearman correlation between ERβ *(ESR2)* and *PROX1* mRNA expression in corresponding TCGA colon tissue. (**C**) Levels of miR-205 is inversely related to PROX1 protein in human colon cancer cell lines SW480, HT29, HCT116, SW403, and SW620. Relative miR-205 levels were determined using qPCR and PROX1 protein levels using western blot. PROX1 protein is low in HT29, but clearly visible when using a longer exposure time (see Figure 3B).

Our previous studies showed that expression of ERβ resulted in increased miR-205 levels [21], and decreased PROX1 levels [23], in SW480 colon cancer cells. To explore the physiological relevance and generality of this proposed regulation, we analyzed RNA-seq data of 233 colon adenocarcinoma and 21 non-tumor colon tissue clinical specimens from The Cancer Genome Atlas (TCGA) dataset. The expression of ERβ in patient samples (Figure 1A, left panel) confirms that in the colon, ERβ expression is decreased in the cancerous state compared to non-cancerous state. In the same data set, miR-205 levels are also reduced in the tumors (Figure 1A, middle panel), while PROX1 levels are increased (Figure 1A, right panel). Furthermore, there was a negative correlation (*p* = 0.0005) between ERβ and PROX1 mRNA levels in clinical colon specimens (Figure 1B). In different human colon cancer cell lines, we observed a clear inverse expression of miR-205 and PROX1 protein (Figure 1C). Corresponding PROX1 mRNA levels are shown in Supplementary Figure S1A. At the mRNA level, the correlation was negative but not significant (*r* = −0.44, *P* = 0.09).

### ERβ upregulates miR-205 in several colorectal cancer cell lines

**Figure 2:**
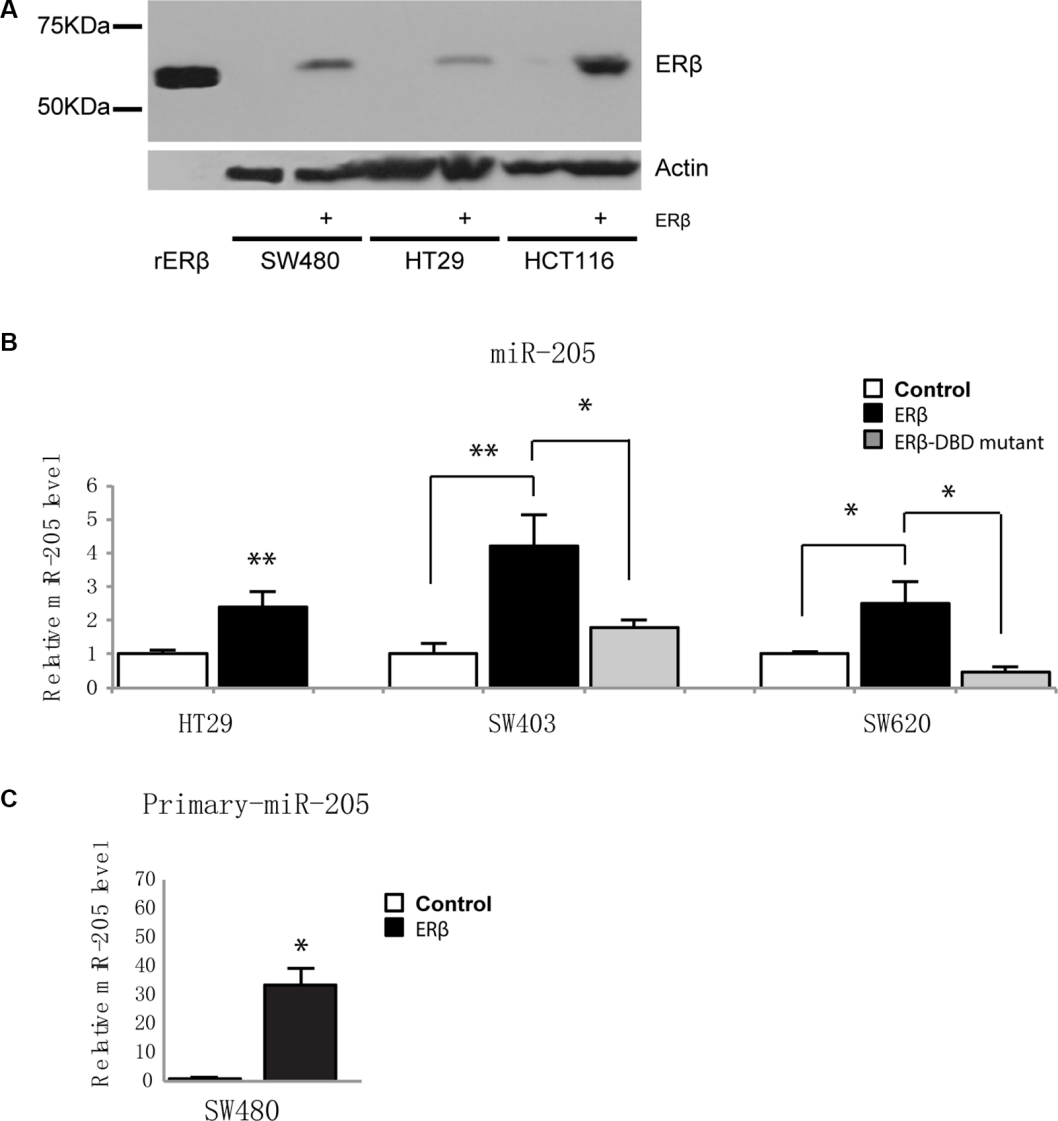
ERβ upregulates miR-205 expression in colon cancer cells (**A**) Western blotting demonstrates FLAG-tagged ERβ protein levels after lentivirus-mediated transduction in SW480, HT29, and HCT116 cells, compared to non-detectable levels in control cells. Recombinant ERβ (59 kDa) was used as positive control (left lane), and β-actin as loading control. (**B**) Mature miR-205 expression is upregulated by ERβ and is dependent on its DNA-binding domain. SW403 and SW620 were transiently transfected by 500 ng pcDNA3.1, ERβ or ERβ-mDBD, and expression determined using miRNA qPCR and normalized to U6 snRNA 48 h after transfection. Transfection and subsequent analysis were replicated three times. (**C**) ERβ regulates primary miR-205 in SW480 cells. Expression normalized to 18S. (*P < 0.05, **P < 0.01, Student's *t*-test).

To investigate whether ERβ can directly increase miR-205 levels, we tested three more cell lines: HT29, SW403 and SW620, before and after re-expression of ERβ. As none of these cell lines express detectable amounts of endogenous ERβ [21, 23] (and Figure 2A), HT29 was stably transduced with ERβ at physiological levels, as previously characterized [21, 23], and SW403 and SW620 were transiently transfected with ERβ plasmid. ERβ upregulated miR-205 in all cell lines (Figure 2B), consistent with our previous observation in SW480 cells. Next, as ERs can bind to cis-regulatory DNA elements either directly through its DNA-binding domain (DBD) or via a tethering mechanism, we tested whether an ERβ mutated in the DBD (ERβ-mDBD) would regulate miR-205. Efficiency of transfection of construct was confirmed using qPCR (Supplementary Figure S1B). ERβ-mDBD failed to increase miR-205 levels in both SW403 and SW620 cells (Figure 2B), suggesting this regulation is dependent on direct DNA binding. Finally, to demonstrate that upregulation of miR-205 is a consequence of transcriptional regulation and not miRNA post-transcriptional processing, we measured the primary transcript (pri-miR-205). We found that pri-miR-205 is strongly elevated by ERβ in SW480 (Figure 2C). The other cell lines had too low levels of this intermediate transcript for robust data. We did not note any effects of E2 treatment on the transcripts (data not shown), supporting the ligand-independent mechanism previously noted upon expression in cell lines [23, 27]. We conclude that ERβ, utilizing its DNA-binding capacity, transcriptionally upregulates miR-205.

### miR-205 directly silences PROX1 by targeting its 3′UTR

**Figure 3:**
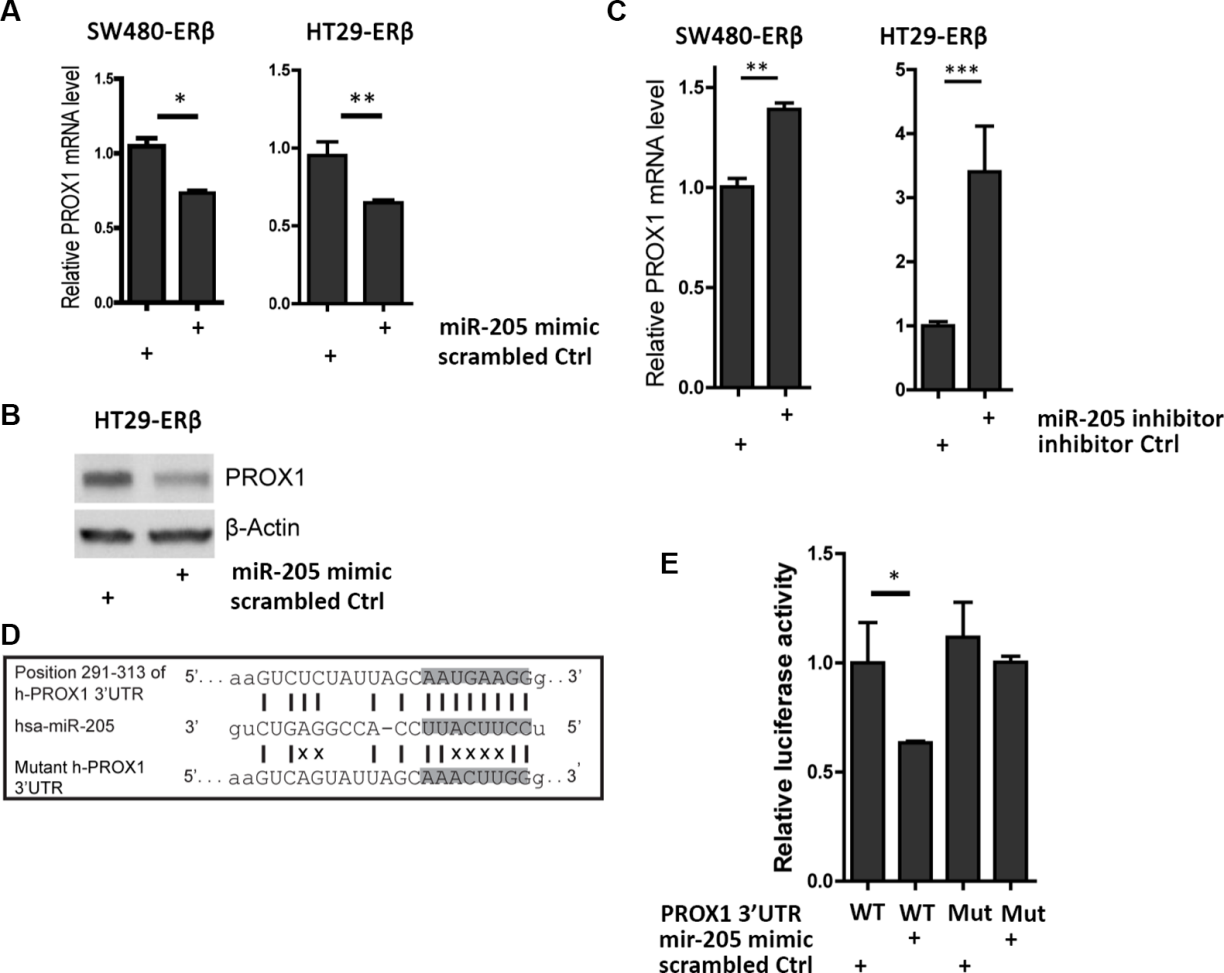
miR-205 directly silences PROX1 by targeting its 3′UTR (**A**) *PROX1* mRNA levels are downregulated by miR-205 overexpression in colon cancer. SW480-ERβ and HT29-ERβ cells were transfected with 50 nM of miR-205 mimic or scrambled mimic control in three replicates, followed by qPCR analysis 48 h after transfection. (**B**) PROX1 protein is repressed by miR-205. Protein was extracted 72 h after single miR-205 mimic or scrambled mimic control transfection and β-actin was used as loading control. (**C**) miR-205 inhibitor upregulates *PROX1* mRNA levels in colon cancer cells. SW480-ERβ and HT116-ERβ cells were transiently transfected by miR-205 inhibitors or inhibitor control at the final concentration of 50 nM, followed by qPCR analysis after 48 h. Experiment replicated two times. (**D**) Sequence alignment between human *PROX1* 3′UTR and mature miR-205 (miRanda). The position refers to distance from the start of 3′UTR. Highlighted nucleotides indicate the seed sequence of miR-205. The mutant human PROX1 3′UTR is represented in the lower panel and the disruption of base-pairing is indicated by X. (**E**) miR-205 directly interacts with the 3′UTR of PROX1. HEK293 cells were co-transfected with wild-type or mutant PROX1 3′UTR luciferase construct (800 ng), and miR-205 mimic or scrambled mimic control (50 nM). Luciferase activity was normalized to Renilla luciferase 24 h after transfection and depicted as the mean ± S.D. The experiment was replicated three times. (*P < 0.05, **P < 0.01, ***P < 0.001, Student's *t*-test).

The inverse relationship between miR-205 and *PROX1* mRNA levels imply that miR-205 may reduce PROX1 expression. To test this, we transfected SW480 and HT29 cells with miR-205 miRNA mimic and measured PROX1 levels. qPCR and western blot showed that PROX1 levels decreased significantly in both cell lines (Figure 3A–3B). Accordingly, miR-205 inhibitors, which block the activity of mature miR-205, increased *PROX1* levels in cell lines that express miR-205 (SW480-ERβ and HCT116-ERβ cells, Figure 3C). To explore whether PROX1 may be a direct target of miR-205, we used miRanda prediction software to scan the 3′UTR of human *PROX1* gene in search of a miR-205 binding site. The 8-nucleotide seed sequence of miR-205 showed complete complementarity to a target site at position 291–313 of the *PROX1* 3′UTR (Figure 3D and Supplementary Figure S2). Using 3′UTR-luciferase-reporter assay we demonstrated that the activity of luciferase cloned with a *PROX1* wild-type 3′UTR was significantly reduced by miR-205 expression, while its activity was unaffected in the clone with a mutation in the putative 3′UTR target site (Figure 3E). Thus, miR-205 repress *PROX1* expression directly through binding to its 3′UTR sequence.

### Tissue-specific knockout of ERβ decreases miR-205 and increases Prox1 in the colon epithelial cells of mice

**Figure 4:**
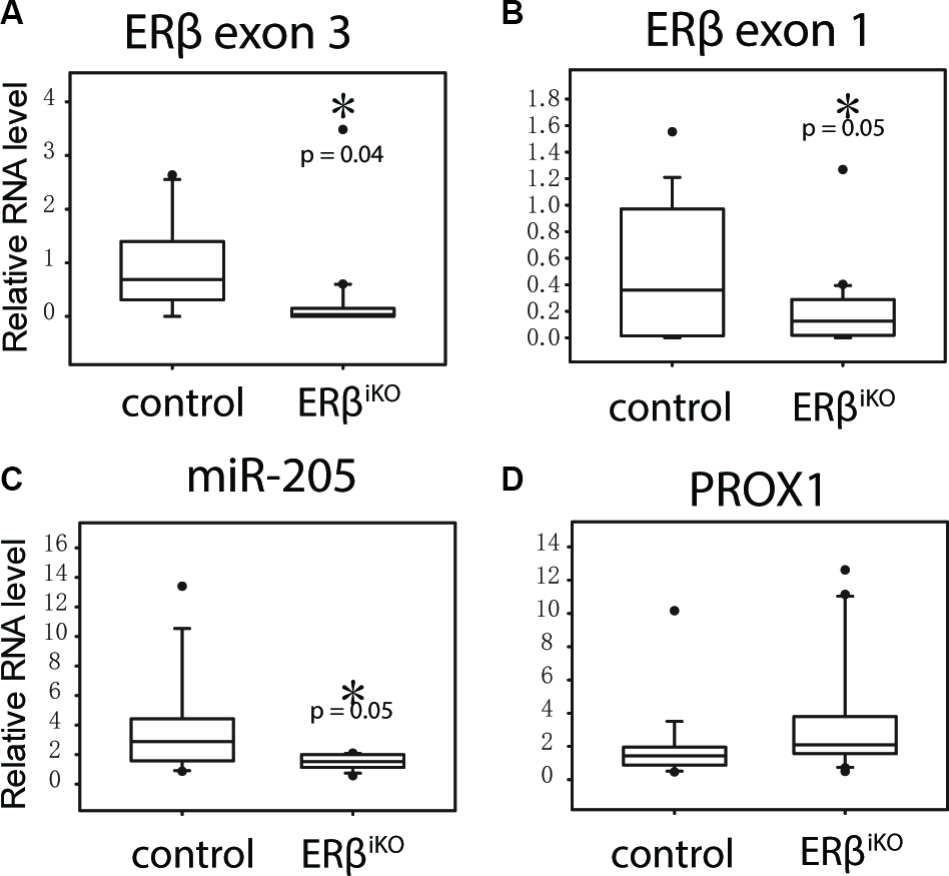
Intestinal-specific KO of ERβ results in decreased miR-205 in colon epithelial cells Colon scraping samples were collected from wild-type mice (*N* = 15) and ERβ^iKO^ (*N* = 16) and RNA expression was analyzed by qPCR. (*P < 0.05, Student's *t*-test). Supplementary Table S1 shows the corresponding correlation between ERβ, miR-205, and PROX1 expression.

To demonstrate that the dysregulation of miR-205 and PROX1 occurs as a result of lost ERβ expression *in vivo*, we generated intestine-specific ERβ knockout mice (ERβ^IKO^). Expression analysis of the deleted ERβ exon 3 in ERβ^IKO^ (*n* = 16) mice compared to controls (*n* = 15) in epithelial intestinal scrape confirmed the knockout (Figure 4A). Expression of exon 1 was also reduced (Figure 4B), presumably through nonsense-mediated mRNA decay, and neither wild-type ERβ nor any residual peptide would be expressed. Levels of miR-205, and *Prox1* were examined using qPCR. We found that as ERβ disappeared, miR-205 levels were also significantly downregulated (*p* = 0.05, Figure 4C), while *Prox1* levels showed a trend of being increased (Figure 4D). As detailed in Supplementary Table S1, expression of both ERβ and miR-205 were inversely correlated with *Prox1* (*p* = 0.037, and *p* = 0.048) while ERβ and miR-205 expression were positively correlated (*p* = 0.028). ERα levels were not affected by the ERβ knockout (Supplementary Figure S1C). Our findings are consistent with the expression data from human clinical samples above, and corroborate the ERβ/miR-205/Prox1 mechanism in normal colon epithelia *in vivo*.

### ERβ and miR-205 reduce cell proliferation

**Figure 5:**
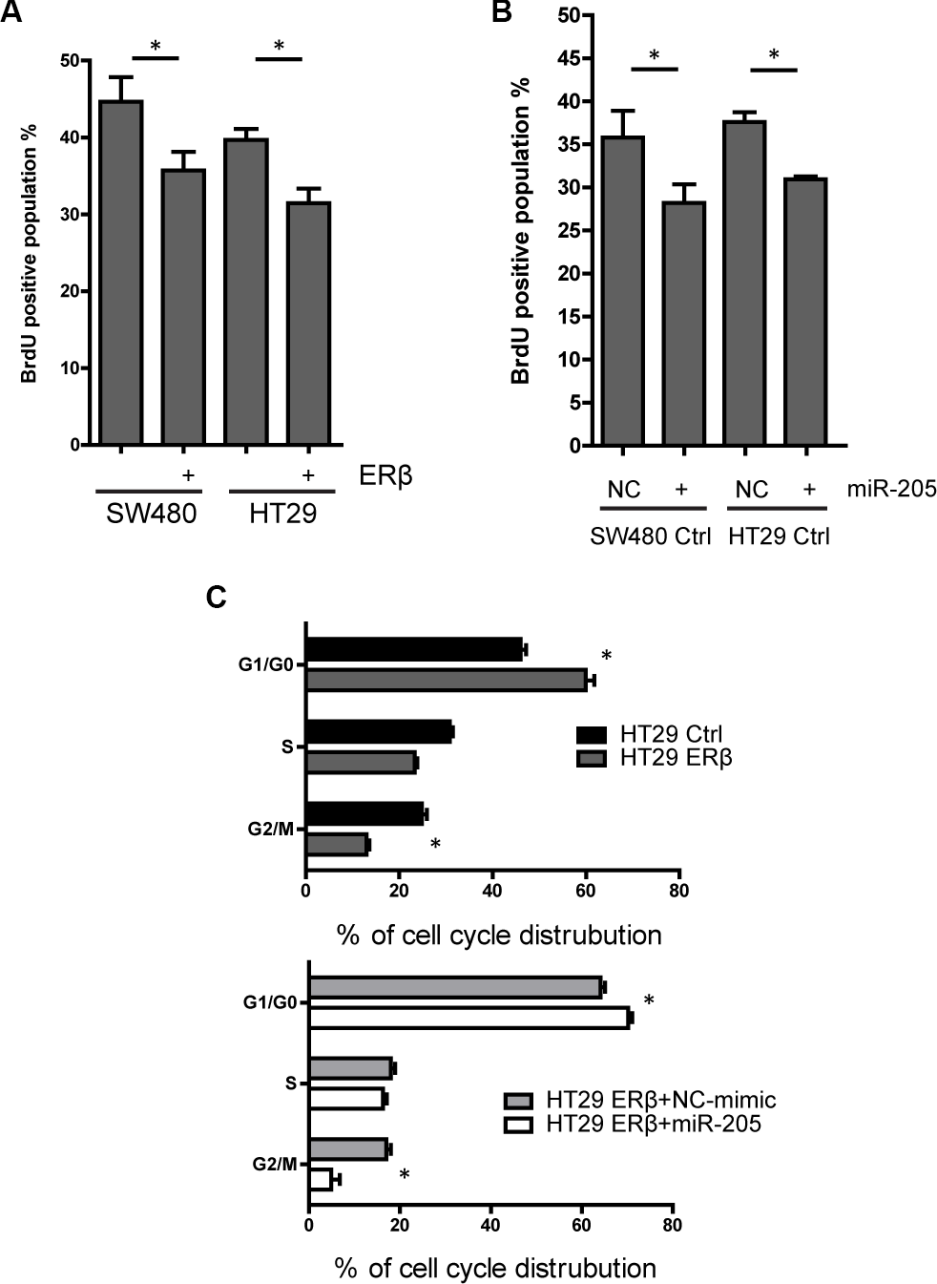
Both ERβ and miR-205 block cell proliferation (**A**) Cell proliferation is inhibited by ERβ expression in SW480 and HT29 cells. BrdU measurment was performed 60 min after BrdU was added to cells engineered to express ERβ and corresponding controls. (**B**) Cell proliferation is repressed by miR-205 transfection. SW480 and HT29 cells were transfected by miR-205 mimic or scrambled control (50 nM, single transfection), followed by BrdU measurment 48 h after transfection. (**C**) Distribution of cell cycle is similarly affected by ERβ and miR-205 overexpression in colon cancer cell lines. Flow cytometry was performed after propidium iodide (PI) staining to assess cell cycle distribution. All experiments were replicated three times. NC: scrambled negative control. (*P < 0.05 compared to control, Student's *t*-test).

Earlier findings, based on using cell counting, have shown that ERβ reduces cell proliferation in SW480 and HCT116 colon cancer cells [23, 27]. We here corroborate that proliferation, measured through incorporation of bromodeoxyuridine (BrdU), were reduced by 24% and 23% in SW480 and HT29 cells upon expression of ERβ (Figure 5A). miR-205 has been reported to affect cell proliferation in different types of cancer [28], but little is known of its effect in colon cancer. We here demonstrate that miR-205 mimic treatment also reduces the BrdU-positive cell population in SW480 and HT29 cells (Figure 5B). Consistently, cell cycle analysis showed that the population of cells remaining in G0 was increased and the population undergoing DNA synthesis and G2/M stage was reduced in a similar manner by either ERβ or miR-205 expression (Figure 5C). Our data show that miR-205 is anti-proliferative in colon cancer cells, and indicate that its regulation contributes to the anti-proliferative effects of ERβ.

### ERβ and miR-205 modulate CD24/CD44 cell populations and adhesion in colon cancer cells

**Figure 6:**
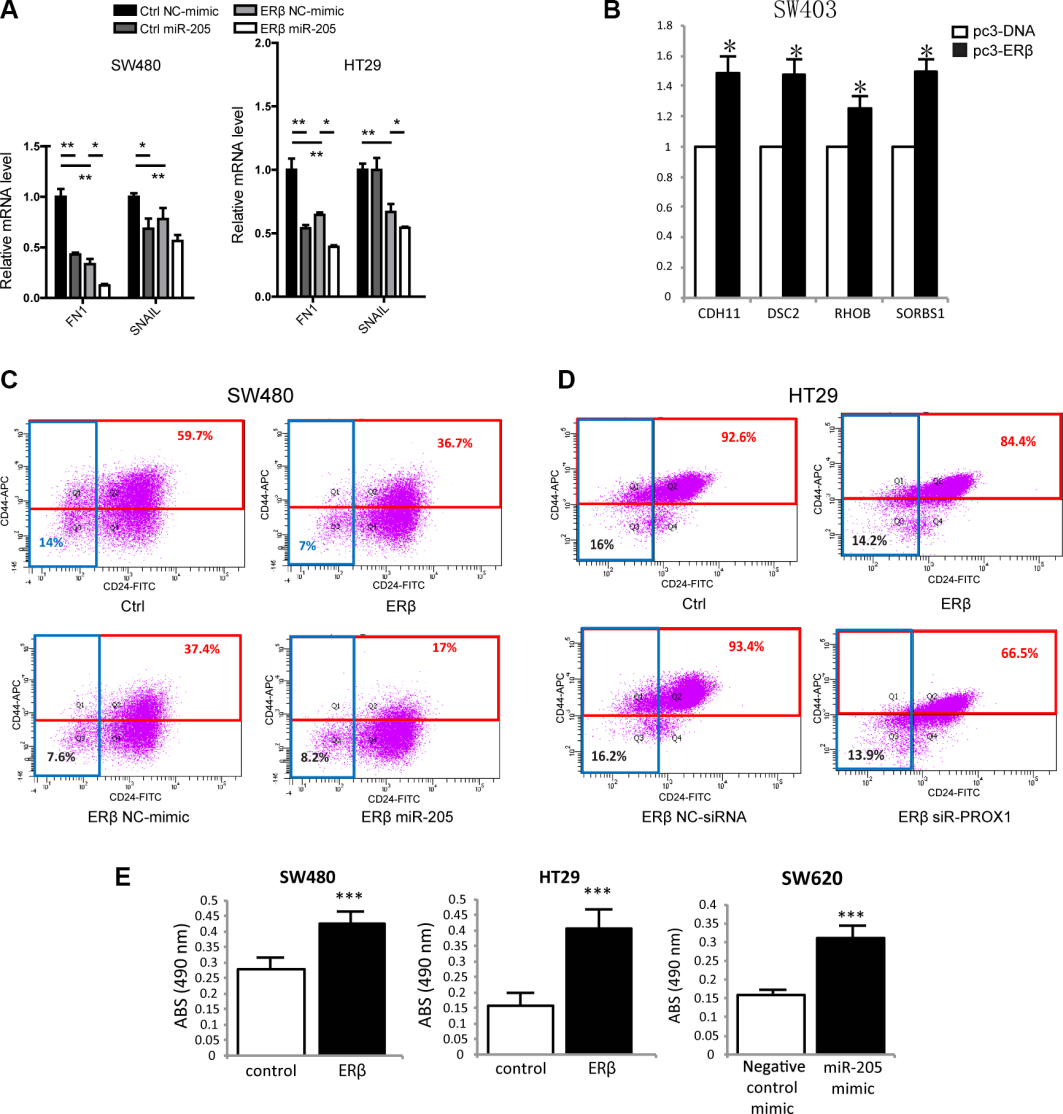
ERβ and miR-205 affects EMT, stemness and cell adhesion in colon cancer (**A**) EMT markers and PROX1-regulated genes FN1 and SNAIL are regulated by both ERβ and miR-205 in SW480 and HT29 cells. mRNA levels were analyzed 48 h after miR-205 double transfection, and experiments were replicated two times. (**B**) Cell adhesion genes are regulated by ERβ in SW403 cells. mRNA levels were analyzed 72 h after ERβ transfection, and experiments were replicated three times. (**C**–**D**) Colon cancer stemness is affected by ERβ, miR-205, and PROX1 in SW480 and/or HT29 cells. Cells were analyzed 48 h after single miR-205 mimic and siR-PROX1 transfection, and experiments were replicated two times. (**E**) ERβ and miR-205 regulate cellular adhesion in several colon cancer cell lines. Cell adhesion assay was performed 72 h after ERβ or miR-205 mimic transfection, and all experiments were replicated three times. Absorbance (Abs) indicates the relative amount of cells that attach to the collagen surface, and is a measure of adhesion. NC: scrambled negative control mimic. (*P < 0.05, **P < 0.01, ***P < 0.001, Student's *t*-test).

Both ERβ and miR-205 have been suggested to regulate epithelial cell differentiation and EMT in different tissues [11, 29]. PROX1 promotes tumor progression in APC^Min/+^ mice, and its knockdown in SW480 colon cancer cells results in increased transcription of genes related to cell adhesion [25]. Therefore, as ERβ and miR-205 repress PROX1, we evaluated whether they impacted expression of different mesenchymal and adhesion markers in colon cancer cells. Stably ERβ-transfected SW480 and HT29 cells showed a reduction of mesenchymal markers FN1 and SNAIL transcripts compared to control cells (Figure 6A). miR-205 mimic treatment (Figure 6A) further reduced their levels, indicating ERβ and miR-205 may attenuate EMT in colon cancer. However, regulation of FN1 and SNAIL was not observed by transient ERβ expression in SW403 or SW620 cells, although in SW403 cells other PROX1-modulated adhesion genes were affected (Figure 6B). Further, as CD24 as well as CD44-positive subpopulations from colon cancer cell-lines have been reported to possess stem cell-like properties [30, 31], we examined effects on this population in SW480 and HT29 cells, using flow cytometry analysis. ERβ decreased the CD44-positive population in SW480 and HT29 cells, miR-205 decreased the CD44-positive population in SW480 cells, and siPROX1 decreased the population in HT29 cells (Figure 6C–6D). The silencing of PROX1 was not efficient at the protein level in SW480 cells, and thus not included. We further measured adhesion capacity using collagen-coated cell plates, and found that expression of ERβ increased cellular adhesion in both SW480 and HT29 cells (Figure 6E). Expression of miR-205 also generated more adhesiveSW620 cells. However, the results were divergent for some experiments: transient expression of ERβ did not increase the adhesion of SW403 or SW620 cells, and we did not notice increased adhesion by miR-205 mimic in SW480 or HT29 cells (data not shown). Thus, while our data are not fully consistent, taken together, it supports that ERβ and miR-205 increase adhesion and therefore, may lessen invasion and/or metastatic potential of colon cancer cells.

### ERβ through miR-205 inhibits cell invasion *in vitro* and *in vivo*

**Figure 7:**
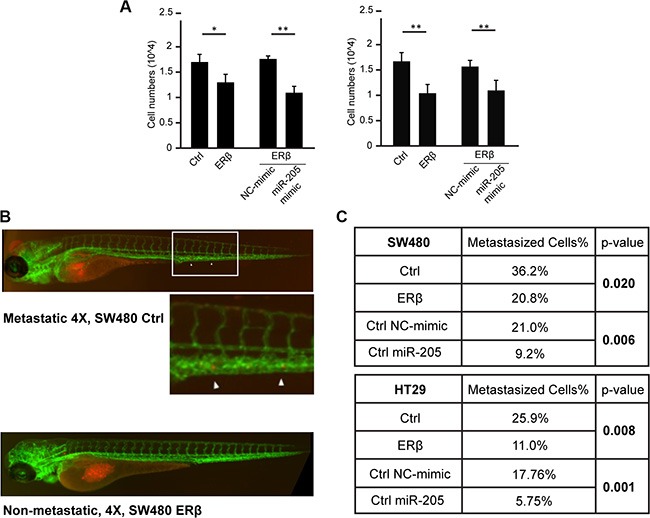
ERβ and miR-205 inhibits tumor invasion *in vitro* and *in vivo* (**A**) Cell invasiveness is reduced by ERβ and miR-205. SW480 and HT29 cells were transfected and transwell assay performed. After 24 h starvation of cells, 10% FBS was used as chemoattractant at the bottom of the chamber. Cells were allowed to migrate for 12 h prior to staining and quantification. (*P < 0.05, **P < 0.01, Student's *t*-test) (**B**–**C**) *In vivo* metastasis assay. Labeled SW480 and HT29 cells, with and without ERβ expression, or with and without transfection of miR-205 mimic, were injected into transgenic *Tg(kdrl:EGFP)mitfa*b692 zebrafish larvae in three independent experiments and observed for metastatic potential after 24 and 48 hpi. Both ERβ and miR-205 exhibited a significant (*p* < 0.05) anti-metastatic potential in both cell lines.

Using transwell invasion assay we found that, consistent with the increase of cell adhesion capacity above and previously reported inhibition of migration in SW480 [21], ERβ significantly reduced cell invasion of both SW480 and HT29 cells (Figure 7A, left). Similar results were noted upon miR-205 transfection (Figure 7A, right). PROX1 knockdown in HT29 cells also reduced invasion as previously reported (data not shown). To evaluate the corresponding impact on *in vivo* metastasis, we used the zebrafish model. We and others have previously shown that the zebrafish organism, *Danio rerio*, is a time-efficient method to evaluate metastatic capacity of different tumor cell lines [32–34]. We injected fluorescence-labeled SW480 and HT29 cells, with and without ERβ expression, and with or without miR-205 mimic, into transgenic zebrafish larvae, and observed the metastatic potential of these cells. As shown in Figure 7B–7C, ERβ exhibited a significant anti-metastatic potential (*p* = 0.02 for SW480 cells and *p* = 0.008 for HT29 cells), reducing the ratio of larvae with metastatic cells compared to larvae without any metastatic cells from 36% to 21% (SW480 cells), and from 26% to 11% (HT29 cells), respectively. Similarly, miR-205 transfection reduced the metastasis potential to only 9% and 6%, respectively (*p* = 0.006 and *p* = 0.001, respectively). Thus, we conclude that ERβ inhibits both invasion and metastatic potential of colon cancer cells through its upregulation of miR-205 *in vitro* and *in vivo*. As PROX1 is known to increase invasiveness [35], its repression by ERβ and miR-205 should be important in relation to colon tumor progression and invasion.

## DISCUSSION

The underlying mechanism whereby ERβ protects against colon cancer development has been largely unclear, but our study details a mechanism by which ERβ simultaneously reduces proliferation and metastatic capacity. We demonstrate that ERβ transcriptionally upregulates miR-205 (Figure 2) and that miR-205, subsequently, represses PROX1 expression through direct interaction with its 3′UTR (Figure 3). Furthermore, as more potential sites are predicted in the 3′UTR of PROX1 (Supplementary Figure S2), miR-205 may interact at multiple sites to regulate PROX1 translation. We validate this relation *in vivo,* showing that tissue-specific ERβ knockouts downregulate miR-205 expression in the luminal surface of the inner colon (Figure 4), and that that expression levels of PROX1 were inversely correlated with ERβ or miR-205 (Supplementary Table S1). We show that in clinical colon specimens and cell lines the expression of ERβ is inversely correlated to PROX1 (Figure 1). Separate studies, based on 643 European colorectal cancer patients, have shown that PROX1 is an important prognostic indicator for colorectal cancer-specific survival [24], further supporting that this regulation is clinically important.

ERβ expression is known to inhibit cell migration in SW480 cells, previously demonstrated through wound healing assays [21]. We here show that ERβ, similar to what has been shown in T47D breast cancer cells [36], increases cellular adhesion of both SW480 and HT29 cells (Figure 6E). The lack of clear phenotype in transiently transfected cells may be due to the shorter time frame of ERβ expression, possibly, this function requires a longer expression to manifest. However, cell adhesion genes previously reported to be repressed by PROX1 in SW480 cells [25], were upregulated as a consequence of transient ERβ expression in SW403 cells (Figure 6B). We show that expression of miR-205 mimic also resulted in increased adhesion (Figure 6E).

In the literature, the role of miR-205 appears cell dependent, as it can act both as an oncogene promoting tumor initiation and growth, or as a tumor suppressor inhibiting cell proliferation and invasion (reviewed in [28]). The role of miR-205 in colorectal cancer is relatively unknown, and miR-205 has been suggested to play dual roles in carcinogenesis [37]. Recent studies have attributed anti-proliferative effects of miR-205 in mammary stem cell fate [38], and in gastric cancer [39]. Here, we show that miR-205 is anti-proliferative in colon cancer cells, and we suggest that this regulation contributes to ERβ's anti-proliferative effect (Figure 5). This function of miR-205 is, however, not dependent on its ability to repress PROX1, as PROX1 does not directly affect the proliferation of colon cancer cells [25].

In metastatic colorectal cancer, miR-205 levels have been reported to be reduced [22], suggesting that its downregualtion is advantageous for metastatic cells. Upon injecting SW480 and HT29 cells into zebrafish embryos we observed clear and consistent anti-metastatic effects of both ERβ and miR-205 *in vivo*. We propose that the upregulation of miR-205 and subsequent repression of PROX1 is important beneficial effects of ERβ activity in the colon, and that the resulting combination of reduced cell proliferation and metastasis is a powerful mechanism. Future experiments should detail the respective contributions' relative impact during colon cancer development and progression.

In conclusion, we have characterized an ERβ-miR-205-PROX1 mechanism *in vitro* and *in vivo.* The functions of ERβ and miR-205, as detailed in this study, reinforce the protective role of ERβ in colon cancer. Thus, selective ERβ agonists may represent a promising strategy for colon cancer chemopreventive therapy. In addition, our results suggest that miR-205 replacement therapy is an avenue to explore in an effort to combat colon cancer metastasis.

## MATERIALS AND METHODS

### Analysis of expression levels in clinical cohort

RNA-seq data from 233 primary colon adenocarcinoma specimens and 21 adjacent normal tissues were downloaded from The Cancer Genome Atlas (TCGA) data portal [40]. RNA-seq by expectation-maximization (RSEM) was used for transcript quantification of mRNAs [41], and reads per million (RPM) where used to quantitate miRNAs. Significance of differences between two groups was determined by two-tailed *t*-test.

### miRNA-205 target prediction

The full-length mRNA sequence of human PROX1 (ENSG00000117707, NM_002763) and mature human miR-205 sequence were obtained from Ensembl and miRBase dataset, respectively. Sequence alignment between PROX1 3′UTR and mature miR-205 was performed using miRanda and Targetscan algorithms.

### Cell culture and transfections

Stable ERβ-expressing cell lines SW480 (CIMP-negative [42]), HT29, and HCT116 (both CIMP-positive [42]) and controls were generated using lentivirus transduction and characterized previously [23]. SW403 and SW620 (purchased from American Type Culture Collection, Manassas, VA, USA) were transfected with wild-type ERβ or ERβ mutated in the DNA-binding domain (ERβ-mDBD), at sites E167A and G168A [27]. These two DBD-mutations abolish the ability of ERβ to bind to an estrogen response element (ERE). SW480 is derived from a primary tumor and SW620 from a lymph node metastasis from the same patient, and the two cell lines carry identical mutation profiles but have epigenetic differences [42]. Cell lines were maintained as previously described [23]. HEK293 cells, used for 3′UTR luciferase assays, were maintained in DMEM supplemented with 10% FBS. Transfection of miRIDIAN mimics, corresponding scrambled control, miRNA hairpin inhibitors, inhibitor control, and ON-TARGET plus siRNA or control (all from Dharmacon, Pittsburgh, PA, USA) were performed as described previously [43], at a final concentration of 50 nM unless otherwise stated. Where noted, 17β-estradiol (E2) treatment at 10 nM concentration was performed to cells after 24 h serum-reduced conditions using dextran-coated charcoal-treated (DCC)-FBS.

### Gene expression analysis

Reverse-transcription quantitative PCR (qPCR) was used to determined changes in transcript levels. Total RNA, including the miRNA population, was extracted using TRIzol (Invitrogen, Grand Island, NY, USA) and miRNeasy spin columns (Qiagen, Valencia, CA, USA) according to the manufacturer's instruction. On-column DNAse I digestion was used to remove remaining genomic DNA. Quantification was performed using NanoDrop 1000 spectrophotometer (Thermo Scientific, Pittsburgh, PA, USA). For miRNA, poly(A) tails were added to 1 μg of total RNA from cell lines or 500 ng of total RNA from mouse tissue samples and cDNA synthesis performed using NCode miRNA First-Strand cDNA Synthesis Kit (Invitrogen) according to the manufacturer's protocol. For qPCR, specific primers for mature miRNA (TCCTTCATTCCACCGGAGTCTG) and precursor-miR-205 (GACAATCCATGTGCTTCTCT) were used. For mRNA and pri-miRNA, 1 μg of total RNA was subjected to cDNA synthesis using SuperScript III First-Strand Synthesis reagents or iScript cDNA Synthesis Kit (Bio-Rad) and random hexamers, according to manufacturer's protocol. qPCR was performed in triplicates using 10 ng of cDNA and iTAq Universal SYBR Green Supermix (Bio-Rad) according to manufacturer's specified conditions, and performed in ABI PRISM 7500 PCR system (Life Technologies). Amplification products were checked with melting curve analysis. Primer sequences are provided upon request. Relative gene expression levels were normalized to 18S, ARGHDIA, and 36B4 for mRNAs and pri-miRNA, and to U6 for miRNAs, and calculated using the ΔΔCT method. Unpaired two-tailed *t*-test was used to test significance between two parallel treatment groups and Pearson test was used for correlation. Results were considered significant if *P* < 0.05.

### Western blot

Cells were washed with PBS, collected, and lysed with RIPA lysis buffer. Protein concentrations were determined using Qubit Protein Assay Kit and Qubit 2.0 Fluorometer (Invitrogen). Approximately 50μg of total protein was resolved on a 10% SDS-PAGE gel, and transferred to nitrocellulose membranes (Bio-Rad, Hercules, CA, USA) according to standard procedure. Membranes were blocked in 10% milk in TBST and incubated with primary antibodies against PROX1 (Upstate, Biotechnology, Lake Placid, NY, 1:1 000 dilution), ERβ (PPZ0506, PPMX, 1:1000 dilution), and β-actin (Sigma-Aldrich, 1:6 000 dilution) overnight, followed by corresponding horseradish peroxidase-linked secondary antibody, and visualized using Pierce ECL western blotting substrate (Thermo Scientific). Scanned images were quantified using ImageJ.

### 3′UTR luciferase assay

Human wild-type or mutant PROX1 3′UTR were cloned downstream of the firefly luciferase gene in the pEZX-MT01 vector (Genecopoeia, Rockville, MD, USA) and co-transfected with miR-205 mimic or control mimic (both from Dharmacon, 30 nM) in HEK293 cells using Lipofectamine 2000 (Invitrogen), according to the manufacturer's protocol. Cells were collected 24 h after transfection and the luciferase activity was examined using Dual-Luciferase reporter assay system (Promega, Madison, WI, USA).

### Proliferation assays

Incorporation of bromodeoxyuridine (BrdU), followed by quantification using flow cytometry analysis, was used to determine proliferation. Cells were starved in 0.5% BSA for 48 h. Transfection, when used, was then performed in regular medium, and 48 h after transfection 30μM BrdU was added to the cells for 60min. Cells were fixed in 70% ethanol and washed with PBS, 2N HCl/Triton X-100, tetraborate and incubated with FITC-conjugated BrdU antibody (BD Biosciences, San Jose, CA, USA) for 30min, followed by analysis using BD FACSAria III (BD Biosciences). Propidium iodide (PI) staining was used to assess cell cycle distribution. Briefly, cells were synchronized in 0.5% BSA and transfected after 48 h in regular medium. After 36 h of proliferation, cells were fixed in 70% ethanol and stained with PI (50 μg/ml, Sigma-Aldrich). The cell cycle distribution of G0/G1, S, and G2/M phase was examined using BD FACSAria III (BD Biosciences).

### Cell fraction analysis

Measurement of CD24/CD44 membrane markers was used to access altered proportion of cells with potential tumor-initiating capacity. Transfection, when used, was performed 48 h prior to analysis. Cells were incubated with CD24-FITC and CD44-APC (both from BD Biosciences) on ice for 15min, resuspended in 0.5 μg/ml PI, and analyzed on BD FACSAria III (BD Biosciences).

### Cell adhesion assay

Transfected cells were trypsinized, counted, and plated on 96-well plates coated with collagen. After 1 h, plates were washed and the amount of attached cells was determined using standard MTS assay (Promega) according to manufacturer's protocol. Plates were read on a SpectroMax M5 Microplate Reader (Molecular Devices, CA, USA).

### Transwell cell invasion assay

Cell invasion capacity was measured using matrigel matrix-coated Boyden chambers (Corning) according to the manufacturer's instruction. Briefly, 48 h following transfection cells were starved in 0.5% BSA medium for 24 h. Upon trypsinization, cells were seeded in chambers and 10% FBS (chemoattractant) was placed in the bottom. After 12 h, cells invading the bottom layer were fixed using 2% formaldehyde, 0.2% glutaraldehyde in PBS, and washed with PBS. Staining with 0.05% crystal violet was performed and ImageJ software was used for quantification.

### Zebrafish micro-metastasis assay

Transgenic zebrafish larvae with green fluorescent protein (GFP)-tagged vascular system (Tg(kdrl:EGFP)mitfa^b692^) were used to study tumor metastasis. SW480 and HT29 cells with and without ERβ and miR-205 mimic were labeled *in vitro* with 2μM of lipophilic dye CM-Dil (Invitrogen) and 500 cells were injected into the yolk of zebrafish larvae at 48 h post-fertilization (hpf), followed by incubation at 32°C. Poorly injected embryos, i.e. direct into blood stream as determined under fluorescent microscope 3 h post injection, were excluded from the study. Tumor cell dissemination in the fish body (mainly in the tail) was monitored using fluorescence microscopy 24 h and 48 h post injection (hpi). Fisher's exact test was used to test the significance of differences in micro-metastasis capacity between the cell lines and treatments.

### Generation of tissue-specific knockout mice and collection of samples

Mice with intestine-specific deletion of ERβ (ERβ^iKO^) were generated by crossing mice with introduced loxP site in introns 2 and 3 of ERβ (B6.129×1-Esr2tm1Gust mice) with mice that express intestinal-specific Cre, driven by the Vilin promoter (Vil-Cre (B6.SJL-Tg(Vil-cre)997Gum/J, Jackson Laboratory, Bar Harbor, Maine). DNA was extracted from an ear punch or tail clipping at 6 weeks of age, and specific primers (provided upon request) were used for genotyping using standard PCR protocol. At 12–20 weeks of age, mice were sacrificed and samples were collected by scraping the epithelial cells of the colon and stored in TRIzol reagent prior to RNA isolation. Lack of ERβ expression was demonstrated comparing mRNA levels for exon 1 and 3 in the scraped epithelial cells of ERβ^iKO^ with controls. Animals were housed in controlled environment at 20°C with illumination schedule of 12 h light, 12 h dark. Standard soy-containg pellet food, which contain phytoestrogens ensuring activity of ERβ, and water were provided *ad libitum*. Animals and procedures in this study were approved by Institutional Animal Care and Use Committee at University of Houston, Houston, Texas.

## SUPPLEMENTARY FIGURES AND TABLES


