# Supplementary material for: Estrogen receptor beta reduces colon cancer metastasis through a novel miR-205 - PROX1 mechanism
Source: Oncotarget. 2016 Jun 7;7(27):42159–71. doi: 10.18632/oncotarget.9895 (PMC5173124; doi:10.18632/oncotarget.9895)
Supplement: Supplementary file 1 [file oncotarget-07-42159-s001.pdf]

Estrogen receptor beta reduces colon cancer metastasis through a novel miR-205 - PROX1 mechanism

Supplementary Materials

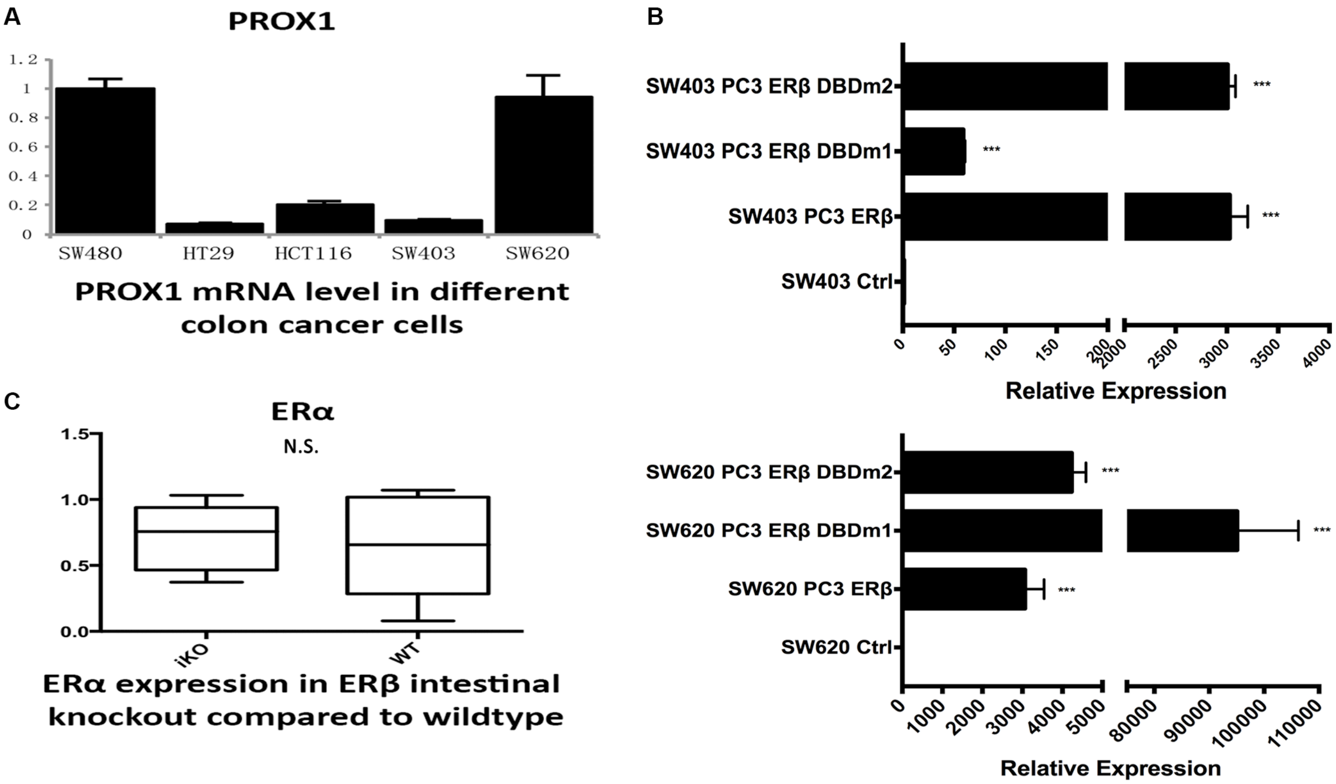

Supplementary Figure S1: mRNA levels measured by qPCR. (**A**) PROX1 in different colorectal cancer cell lines. The inverse correlation between miR-205 and PROX1 mRNA levels ($r = -0.44$) were not significant ($p = 0.09$). (**B**) Expression of transiently transfected ERβ and ERβ-DBD mutant corroborated in SW403 and SW620 cell lines. (*$P < 0.05$, **$P < 0.01$, Student's *t*-test). C. Expression of ERα in colon epithelial scrape from intestine-specific ERβ knockout mice (iKO) and corresponding controls (WT) is not changed.

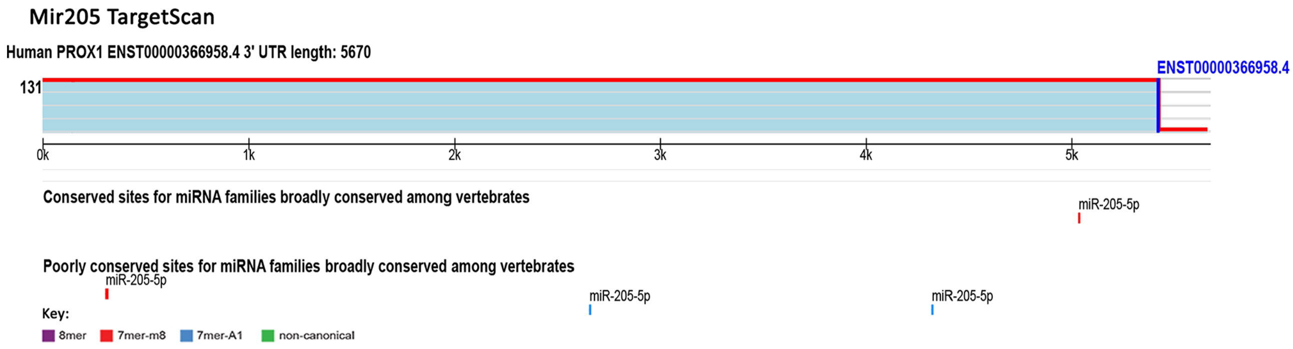

Supplementary Figure S2: Predicted miR-205 sites of PROX1 3′UTR using Targetscan. In this study we demonstrate that the site at position 291-313 nt 3′ of the coding sequence (to the left in figure) *de facto* interacts with miR-205.

Supplementary Table S1: Correlation between ERβ, Prox1, and miR-205 in ERβ^{iKO} mouse colon tissue

	PROX1	miR-205
ERβ	−0.38 (p = 0.037)	0.51 (p = 0.028)
PROX1		−0.05 (p = 0.048)

Expression of ERβ is correlated to miR-205 expression, while inversely correlated to *Prox1* expression. Correlations were analyzed using Pearson correlation, and considered significant if $p < 0.05$.
